# Pilot randomised controlled trial of culturally adapted cognitive behavior therapy for psychosis (CaCBTp) in Pakistan

**DOI:** 10.1186/s12913-017-2740-z

**Published:** 2017-12-06

**Authors:** Muhammed Omair Husain, Imran B. Chaudhry, Nasir Mehmood, Raza ur Rehman, Ajmal Kazmi, Munir Hamirani, Tayyeba Kiran, Ameer Bukhsh, Paul Bassett, Muhammad Ishrat Husain, Farooq Naeem, Nusrat Husain

**Affiliations:** 1 Faculty of Medical and Human Sciences, University of Manchester, Room G.907, Stopford Building, Oxford Road, Manchester, M13 9PT UK; 2 Pakistan Institute of Learning and Living, Karachi, Pakistan; 3 Dow University of Health Sciences, Karachi, Pakistan; 4 Abbasi Shaheed Hospital, Karachi, Pakistan; 5 Statsconsultancy Ltd, Amersham, UK; 6 Camden and Islington NHS Foundation Trust, London, UK; 7 Queens’ University, Kingston, ON Canada

**Keywords:** Culture, Cognitive behavioural therapy, Pakistan, Psychosis, Schizophrenia

## Abstract

**Background:**

Evidence for efficacy of cognitive-behavioural therapy (CBT) in treatment of schizophrenia is growing. CBT is effective and cost efficient in treating positive and negative symptoms. To effectively meet the needs of diverse cultural groups, CBT needs to be adapted to the linguistic, cultural and socioeconomic context. We aimed to assess the feasibility, efficacy and acceptability of a culturally adapted CBT for treatment of psychosis (CaCBTp) in a low-income country.

**Methods:**

Rater-blind, randomised, controlled trial of the use of standard duration CBT in patients with psychosis from a low-income country. Participants with a ICD-10 diagnosis of psychosis were assessed using Positive and Negative Syndrome Scale for Schizophrenia (PANSS), Psychotic Symptom Rating Scales (PSYRATS), and the Schedule for Assessment of Insight (SAI) (baseline, 3 months and 6 months). They were randomized into the intervention group (*n* = 18) and Treatment As Usual (TAU) group (*n* = 18). The intervention group received 12 weekly sessions of CaCBTp.

**Results:**

The CaCBTp group had significantly lower scores on PANSS Positive (*p* = 0.02), PANSS Negative (*p* = 0.045), PANSS General Psychopathology (*p* = 0.008) and Total PANSS (*p* = 0.05) when compared to TAU at three months. They also had low scores on Delusion Severity Total (*p* = 0.02) and Hallucination Severity Total (*p* = 0.04) of PSYRATS, as well as higher scores on SAI (*p* = 0.01) at the same time point. At six months only the improvement in PANSS positive scores (*p* = 0.045) met statistical significance..

**Conclusions:**

It is feasible to offer CaCBTp as an adjunct to TAU in patients with psychosis, presenting to services in a lower middle-income country.

**Trial registration:**

Clinicaltrials.gov identifier NCT02202694 (Retrospectively registered).

## Background

Schizophrenia is the most common psychotic disorder worldwide, affecting over 26 million people [1]. Data from meta-analysis has demonstrated that antipsychotic medication is effective in reducing risk of relapse in schizophrenia [2]. However, it has been reported that 25–50% of patients with psychosis continue to experience distressing symptoms despite compliance with neuroleptic medication [3]. Non-compliance as a result of intolerable side effects of neuroleptics greatly increases rates of relapse [4]. The last two decades have seen advances in the development of effective non-pharmacological treatments for psychotic disorders.

Cognitive Behavioural Therapy (CBT) has been found to be effective in improving positive and negative symptom severity in schizophrenia [5, 6]. A recent meta-analysis reported that CBT had a therapeutic benefit in treating symptoms of schizophrenia, albeit with a small effect size [7]. It is crucial to note that when controlling for biases, such as masking, these effect sizes became insignificant, particularly in the case of positive symptoms. A more recent review, identified the value of CBT in improving mental state, with reduction in positive symptoms, in particular command hallucinations [8]. There is also RCT evidence that brief CBT delivered by mental health nurses is effective in reducing the severity of psychotic symptoms [9]. In the UK, the National Institute for Health and Care Excellence (NICE) recommends offering CBT in conjunction with anti-psychotic medicine to all patients, for the treatment of schizophrenia [10].

CBT is widely used in the high-income countries for treating psychiatric disorders, but the use of psychological interventions is very limited in lower-middle income countries, like Pakistan. Recent evidence from low and middle-income countries, suggests beneficial effects of psychological interventions for psychiatric disorders [11–14]. Social, religious and cultural factors influence the perception of mental illness, in turn impacting health-related behaviour and engagement with services. These factors need to be taken in to account when tailoring psychological therapies to a specific group [15]. Evidence from systematic reviews has suggested that cultural adaptations of interventions can have a moderately strong benefit on efficacy [16, 17]. A 2006 meta-analysis of culturally adapted mental health interventions found that that racial composition of the participants impacted efficacy. Interventions targeting specific groups were more effective than those offered to participants from various ethnic and cultural backgrounds [16]. Delivering mental health interventions in the native language (if other than English) of participants, resulted in a two-fold increase in effectiveness [16]. A very recent meta-analysis drew similar conclusions; that culturally adapted psychosocial interventions were more efficacious (with moderate effect sizes) in treating symptoms of schizophrenia, when compared to usual treatment [17]. Furthermore, the degree of adaptation was directly proportional to efficacy [17]. It must be noted that the authors could not conclusively determine if culturally adapted interventions were more effective than those that were not culturally adapted. Evidence suggests there is a role for culturally adapted CBT in the treatment of psychotic disorders in some black and minority ethnic groups [18].

Our group recently adapted CBT, in a brief form (6 to 10 sessions), as adjunctive treatment in patients with psychosis [19]. We are aware that cultural and religious factors, cognitions and illness beliefs, and available resources need to be considered when adapting CBT. We found that culturally adapted, brief CBT for psychosis was feasible as an adjunct to treatment as usual. The recruitment and retention rates were excellent, giving weight to the acceptability of this therapy to patients. This current study is primarily intended to assess the feasibility of standard duration (12–16 sessions), culturally adapted CBT for the treatment of psychosis (CaCBTp) in a low-income country. Recruitment, retention and adherence to the intervention will inform “feasibility”. Furthermore, we will assess the efficacy of the intervention in treating symptoms of psychosis.

## Methods

### Research design

This was a 12-week pilot trial using Randomized Control Trial (RCT) design, in which CaCBTp added on to Treatment as Usual (TAU) was compared to TAU. Patients with a diagnosis of schizophrenia spectrum disorders (ICD 10 codes F20–29) who were attending outpatient psychiatric services in Karachi, Pakistan, were included.

### Sample

A total of 36 participants were recruited from psychiatric departments of different hospitals, in Karachi, Pakistan. They were randomly divided into two groups; CaCBTp group and treatment-as-usual group (TAU). This was to ensure that, even after the loss to follow-up, we would have at least 12 subjects per group for analysis. Recommendations regarding sample sizes have been informed from prior studies, which suggest 24 to 50 participants for a pilot trial [20, 21]. Randomization was carried out by an offsite statistician. A simple 1:1 randomization was carried out using, http://www.randomization.com. A total of 18 subjects were allocated into each group, making a total of 36 participants in both groups. For the intervention group, twelve sessions of CaCBTp were provided by a trained research clinician, over a three-month period. Patients in the TAU group continue to receive their routine treatment.

### Recruitment of patients and procedure

All consecutive patients with a diagnosis of psychotic disorder referred to research clinicians by outpatient department doctors were recruited. The participants were not paid to take part in the study, however, they were given travel expenses. The initial data was gathered by research clinicians and those patients who fulfilled the inclusion criteria for the study were registered. All participants provided written informed consent. The participants who consented to take part in the pilot were then randomly allocated to one arm of the trial. Stratification procedures were not in place, therefore, there was an unequal spread of participants from different sites. Participants were interviewed by a ‘blind rater’ at baseline and the end of the intervention. The raters had received training in the use of scales and were psychology graduates. To maintain blinding, raters carrying out assessments were not given any information regarding allocation of participants to treatment groups. Furthermore, participants were asked not to disclose any information regarding the treatment they had received.

### Inclusion criteria

1. Diagnosis of psychosis established by the clinicians using ICD10 criteria (including schizophrenia, schizoaffective disorder, or delusional disorder using ICD-10 research criteria).
2. Individuals aged 18–65 years.
3. Being a resident of Karachi.
4. The participant could give informed consent (written).

### Exclusion criteria

1. Severe illness which could impair capacity or significantly impact the participant’s ability to engage in an interview, e.g. very thought disordered or distressed by symptoms.
2. Those who fulfil the criteria for substance dependence according to ICD10 criteria.
3. Diagnosed intellectual impairment.
4. Presence of organic cognitive impairment

### Aims and objectives

This study primarily aimed to establish the feasibility of standard 12 sessions CaCBTp. “Feasibility” was informed by recruitment and retention rates. The drop-out rates and attendance data informed “acceptability” of the intervention.

Furthermore, we aimed to evaluate the impact of the intervention on overall Positives and Negative Syndrome scale (PANSS) score [22], Psychotic Symptom Rating Scales (PSYRATS) [24], Schedule for Assessment of Insight (SAI) [25], and the Calgary Depression Scale for Schizophrenia (CDSS) [26]. The PANSS is measured on a 7-point scale, and is a 30-item structured clinical interview assessing symptom severity over the previous week [22]. There are three subscales, PANSS Positive Subscale (7 items measuring positive symptoms), PANSS Negative Subscale (7 items measuring negative symptoms) and PANSS General Psychopathology Subscale (16 item measuring general symptoms that are associated with schizophrenia) [22]. PSYRATS has 17 items, measured on a scale of 0–4, exploring specific characteristics of hallucinations and delusions [23]. Higher scores on the PANSS and PSYRATS indicate higher severity of illness. The SAI measures three aspects of insight related to treatment adherence, symptom relabelling and illness recognition. The cumulative scores from these domains give a total insight score, with higher scores indicating greater insight [24]. The CDSS was specifically designed to assess depression in patients with schizophrenia [25]. The scale has 9 items scored from 0 to 3, with higher scores suggestive of higher severity [25]. All scales were translated into Urdu and have been used in previous studies in Pakistan [19]. There is limited evidence on the cross-cultural validity of these scales in Pakistan [26].

### Intervention

Participants in the CaCBTp group received 12 individual CaCBTp sessions from a trained research psychologist, who was trained in delivering CaCBTp. The individual needs of the participant were accommodated with flexibility in duration and frequency of the sessions. However, the aim was to offer at least forty-five minutes of therapy, once a week during the three-month period. Failure to engage was defined as attendance at less than six therapy sessions. The participants in this group continued treatment as usual, alongside the intervention.

The CaCBTp intervention is based on the treatment manual developed by David G. Kingdon and Douglas Turkington [27], and culturally adapted by author FN. CaCBTp aims to take a collaborative approach to gaining an understanding of the symptoms experienced, working towards reducing distress and disability. There are distinct stages, including engagement, the examination of antecedents of the emerging psychotic disorder, the development of normalising rationale, the treatment of co-morbid anxiety or depression, and collaboratively constructing a case formulation. CaCBTp uses specific techniques for positive symptoms of schizophrenia thereafter. For addressing auditory hallucinations, beliefs about the origin and nature of the experiences(s) are explored using collaborative critical analysis. Strategies such as voice diaries, reattributing the cause of the voices, and development of coping strategies are also employed. Guided discovery and graded homework tasks are used to elucidate delusions. Focusing on specific themes, clarification of neologisms, and thought linkage are some of the techniques used to improve thought disorder. After work on positive symptoms, negative symptoms are targeted using activity scheduling and records of mastery and pleasure in a diary.

### Cultural adaptation of the intervention

We have experience in adapting interventions for self-harm, depression and psychosis in Pakistan and the UK. Our group has culturally adapted interventions for depression, self-harm and psychosis using mixed methods, in Pakistan and the UK [13, 19, 28]. With regards to psychological interventions, the adaptation is generally focused on implementation, rather than the content of the treatments [29]. With the CaCBTp, the same principles were adhered to. In CaCBTp culturally acceptable idioms were used to explain concepts related to the symptoms and causes of psychotic disorder. For example, to explain the concept of multiple perspectives, information was drawn from religious teachings, as well as local stories and images. To improve participant engagement, simple strategies were implemented. In our experience, using culturally appropriate terminology, speaking in the native tongue (Urdu) and aiming to build rapport during sessions, has been beneficial. Carers were invited to take part in sessions, if permission was granted by the participants, to help build a trusting relationship during the sessions.

### Treatment-as-usual

The participants in this group continued treatment as usual (care continued to be provided in outpatient clinic at regular intervals and the participants continued to adhere to medication as prescribed by their responsible clinical team) and were compared with CaCBTp group after three and six months.

### Assessments

Independent assessors blind to the randomization status of the participants carried out assessments after completion of the intervention (three months). All participants were reassessed at six months to assess the longer-term effects of CaCBTp. An interview schedule was developed to enable scoring of the PANSS and SAI to be completed simultaneously. These measures were completed by the research clinician at baseline, after randomization, three months and six months.

### Statistical analysis

Analyses were performed using SPSSv16 for Windows (SPSS Inc., Chicago). One of the aims of this pilot study was to provide estimates of effect size to inform a full study of the intervention in the future. Intention to treat (ITT) analysis was performed on all outcome measure scores post therapy. The outcome of the two groups was evaluated using analysis of covariance. In the analyses, the follow-up (T2 or T3) timepoint is treated as the outcome variable, and the covariate is the equivalent variable at baseline (T1). Secondary continuous outcomes were analysed using ANCOVA. Some of the categorical baseline variables were analysed using Fisher’s exact test. All continuous outcome variables were checked for the assumption of normality using Kolmogorov Smirnoff test.

The study is reported in accordance with the CONSORT (Consolidated Standards of Reporting Trials) statement.

## Results

The socio-demography and baseline symptom severity, showed no statistically significant differences when comparing groups (see Tables 1 and 2). The sample in both groups was predominantly male (77.8% in CaCBTp and 55.6% in TAU group). There were marginally less single persons in the CaCBTp (72.2%) group, compared with the TAU group (77.8%). The CaCBTp group had higher rates of unemployment (66.7%) than TAU (44.4%). In both groups, only 5.6% of participants had over three previous hospital admissions.

**Table 1:** Baseline characteristics by treatment group and overall

Baseline characteristics	Treatment group		Total (*N* = 36)
	CBT (*N* = 18)	TAU (*N* = 18)	
Age – mean (SD)	34.1 (9.55)	30.5 (8.15)	32.3 (8.94)
Number of years education – mean (SD)	8.9 (4.86)	7.8 (4.40)	8.4 (4.60)
Gender			
Male	14 (77.8%)	10 (55.6%)	24 (66.7%)
Female	4 (22.2%)	8 (44.4%)	12 (33.3%)
Marital status			
Single	13 (72.2%)	14 (77.8%)	27 (75.0%)
Married	4 (22.2%)	4 (22.2%)	8 (22.2%)
Divorced	1 (5.6%)	0 (0.0%)	1 (2.8%)
Employment			
Employed	6 (33.3%)	8 (44.4%)	14 (38.9%)
Unemployed	12 (66.7%)	8 (44.4%)	20 (55.6%)
House wife	0 (0.0%)	2 (11.1%)	2 (5.6%)
Total number of prior inpatient psychiatric hospitalisations			
None	11 (61.1%)	9 (50.0%)	20 (55.6%)
Once	0 (0.0%)	2 (11.1%)	2 (5.6%)
Twice	3 (16.7%)	2 (11.1%)	5 (13.9%)
Thrice	3 (16.7%)	4 (22.2%)	7 (19.4%)
4 or more	1 (5.6%)	1 (5.6%)	2 (5.6%)

For continuous baseline characteristics (age and number of years of education) an independent t-test was used to test for differences between treatment groups. For categorical baseline characteristics (gender, marital status and employment), the Fisher’s exact test was used to test for differences between treatment groups. For the total number of prior inpatient psychiatric hospitalisations, since this data was ordinal then the Wilcoxon-Mann Whitney U test was used to test for differences between treatment groups

Baseline characteristics appear to be fairly balanced across the two treatment groups with no statistically significant differences between CBT and TAU treatment groups

**Table 2:** Baseline outcomes by treatment group

Outcome	Treatment group	
	CaCBTp (*N* = 18)	TAU (*N* = 18)
	Mean (SD)	Mean (SD)
Baseline: Sub-Total of Positive Subscale (PANSS)	14.61 (6.98)	14.50 (4.93)
Baseline: Sub-Total of Negative Subscale (PANSS)	14.83 (4.29)	14.61 (3.87)
Baseline: Sub-Total of General Psycho-pathology Subscale (PANSS)	31.94 (10.86)	31.83 (7.69)
Baseline: Final Total of PANSS	61.39 (19.31)	60.94 (14.17)
Baseline Delusion Severity Total	7.89 (7.00)	9.78 (6.90)
Baseline Hallucination Severity Total	10.5 (13.65)	8.33 (13.23)
Baseline SAI Total	8.89 (4.04)	8.00 (3.99)
Baseline Depression Total	6.39 (4.53)	6.61 (4.80)

### Feasibility: Recruitment and retention

**Fig. 1:**
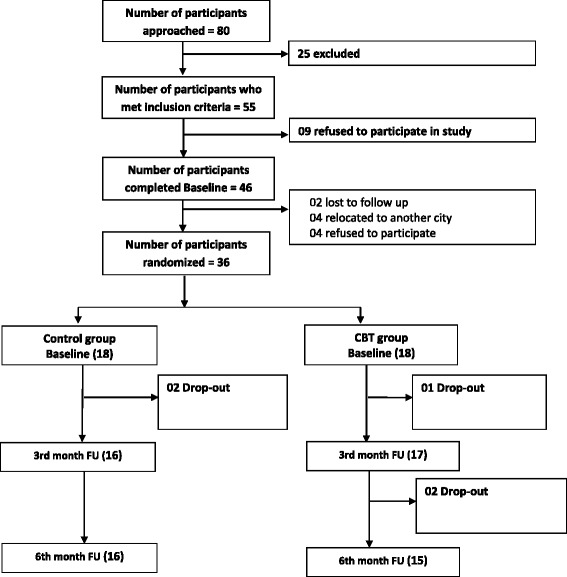
Consort flow chart

Feasibility of the intervention was inferred from the excellent recruitment and retention rates. Figure 1 summarizes participant flow through the study, illustrating the successful recruitment of the intervention. 80 participants were found to be suitable for recruitment. 55 of the initial 80 participants approached, met inclusion criteria. 83% of participants who met inclusion criteria, were recruited with 10 unwilling to take part. Subsequently, 36 patients were randomized.

Most of the participants who were randomised, completed the study (*n* = 31). Therefore, the retention rates within the study were an encouraging finding (86%). 2 of the 18 participants randomised to the TAU group, were lost to follow up. Similarly, out of the 18 randomised to the CaCBTp group, 3 participants dropped out. Unfortunately, these participants were also lost to follow up. The CaCBTp intervention was acceptable considering that more than 80% of participants attended 9–12 sessions. We have inferred acceptability of the intervention from the high rates of attendance.

The mean and standard deviation (SD) for the outcomes at 3 months (T2) and 6 months (T3) are presented in Tables 3 and 4. Analysis of covariance (ANCOVA) models was used to assess for differences in outcomes at T2 and T3 between the CaCBTp and TAU treatment groups adjusting for the baseline outcome. Separate ANCOVA models were fitted for each outcome at T2 and at T3. Adjusted means and 95% confidence intervals (CIs) are presented for each treatment group and for the difference between the two treatment groups.

**Table 3:** Time point 2 (T2) outcomes by treatment group

Outcome	Statistics	Treatment group		Adjusted mean difference (95% CI) (CaCBTp – TAU)	Effect size	*P*-value
		CaCBTp (*N* = 17)	TAU (*N* = 16)			
Sub-Total of Positive Subscale (PANSS)	Raw mean (SD)	12.88 (4.01)	17.31 (7.34)			
	Adjusted mean (95% CI)	12.76 (10.05, 15.46)	17.45 (14.66, 20.24)	−4.69 (−8.58, −0.80)	0.32	0.02
Sub-Total of Negative Subscale (PANSS)	Raw mean (SD)	13.18 (3.70)	16.38 (6.13)			
	Adjusted mean (95% CI)	13.02 (10.63, 15.40)	16.55 (14.08, 19.01)	−3.53 (−6.97, −0.09)	0.24	0.045
Sub-Total of General Psycho-pathology Subscale (PANSS)	Raw mean (SD)	27.53 (6.76)	34.69 (8.49)			
	Adjusted mean (95% CI)	27.36 (23.62, 31.09)	34.87 (31.02, 38.72)	−7.51 (−12.88. -2.14)	0.26	0.008
Final Total of PANSS	Raw mean (SD)	53.59 (12.46)	68.38 (19.11)			
	Adjusted mean (95% CI)	53.03 (45.55, 60.51)	68.97 (61.25, 76.68)	−15.94 (−26.71, −5.17)	0.51	0.005
Delusion Severity Total	Raw mean (SD)	6.82 (5.93)	11.69 (6.97)			
	Adjusted mean (95% CI)	6.99 (4.33, 9.65)	11.51 (8.77, 14.25)	−4.52 (−8.34, −0.70)	0.71	0.02
Hallucination Severity Total	Raw mean (SD)	5.59 (9.25)	11.19 (15.23)			
	Adjusted mean (95% CI)	5.05 (0.52, 9.59)	11.75 (7.08, 16.43)	−6.70 (−13.22, −0.18)	0.43	0.04
SAI Total	Raw mean (SD)	11.00 (3.84)	7.13 (4.96)			
	Adjusted mean (95% CI)	10.87 (9.00, 12.74)	7.26 (5.33, 9.19)	3.61 (0.92, 6.30)	0.16	0.01
Depression Total	Raw mean (SD)	3.53 (3.50)	4.56 (4.94)			
	Adjusted mean (95% CI)	3.51 (1.57, 5.46)	4.58 (2.58, 6.59)	−1.07 (−3.87, 1.72)		0.44

**Table 4:** Time point 3 (T3) outcomes by treatment group

Outcome	Statistics	Treatment group		Adjusted mean difference (95% CI) (CaCBTp – TAU)	Effect size	*P*-value
		CaCBTp (*N* = 15) Adjusted mean (95% CI)	TAU (*N* = 16) Adjusted mean (95% CI)			
Sub-Total of Positive Subscale (PANSS)	Raw mean (SD)	13.80 (4.20)	17.31 (5.75)			
	Adjusted mean (95% CI)	13.70 (11.10, 16.30)	17.41 (14.89, 19.93)	−3.71 (−7.34, −0.09)	0.25	0.045
Sub-Total of Negative Subscale (PANSS)	Raw mean (SD)	14.27 (2.31)	14.69 (2.50)			
	Adjusted mean (95% CI)	14.18 (13.00, 15.37)	14.77 (13.62, 15.91)	−0.58 (−2.23, 1.07)	0.04	0.47
Sub-Total of General Psycho-pathology Subscale (PANSS)	Raw mean (SD)	28.47 (4.60)	30.44 (6.45)			
	Adjusted mean (95% CI)	28.36 (25.38, 31.33)	30.54 (27.66, 33.42)	−2.18 (−6.33, 1.97)	0.07	0.29
Final Total of PANSS	Raw mean (SD)	56.53 (9.71)	62.44 (12.87)			
	Adjusted mean (95% CI)	56.24 (50.27, 62.21)	62.71 (56.93, 68.49)	−6.47 (−14.80, 1.86)	0.11	0.12
Delusion Severity Total	Raw mean (SD)	8.80 (7.06)	12.31 (6.63)			
	Adjusted mean (95% CI)	8.83 (5.52, 12.14)	12.28 (9.08, 15.49)	−3.45 (−8.06, 1.15)	0.39	0.14
Hallucination Severity Total	Raw mean (SD)	13.53 (13.54)	9.25 (14.96)			
	Adjusted mean (95% CI)	13.36 (5.68, 21.04)	9.41 (1.98, 16.85)	3.95 (−6.78, 14.68)	0.42	0.46
SAI Total	Raw mean (SD)	10.40 (4.10)	7.75 (4.74)			
	Adjusted mean (95% CI)	10.14 (7.97, 12.32)	7.99 (5.88, 10.10)	2.15 (−0.89, 5.20)	0.25	0.16
Depression Total	Raw mean (SD)	6.27 (4.01)	4.69 (3.66)			
	Adjusted mean (95% CI)	6.25 (4.19, 8.32)	4.70 (2.70, 6.70)	1.55 (−1.32, 4.43)	0.24	0.28

Apart from the outcome Depression Total (*p* = 0.44) all other outcomes at time T2 were significantly different between the two treatment groups. Patients in the CaCBTp treatment group had significantly lower scores compared to patients in the TAU treatment group for the outcomes: Sub-Total of Positive Subscale (*p* = 0.02) Sub-Total of Negative Subscale (*p* = 0.045), Sub-Total of General Psycho-Pathology Subscale (*p* = 0.008), Final Total of PANSS (*p* = 0.005), Delusion Severity Total (*p* = 0.02) and Hallucination Severity Total (*p* = 0.04). Whereas patients in the CaCBTp treatment group had significantly higher scores compared to patients in the TAU treatment group for the outcome SAI (*p* = 0.01).

At T3, there are no significant differences in outcomes between the two treatments groups apart from the outcome Sub-Total of Positive Subscale (*p* = 0.045). For Sub-Total of Positive Subscale patients in the CaCBTp group had a significantly lower score compared to patients in the TAU treatment group.

## Discussion

There is limited published literature exploring the use of CBT in psychosis for patients from Lower-middle-Income countries. The results confirm findings from our previous study in which low intensity CBT was tested in Pakistan [19]. Our current study used a similar methodology, and aimed to assess the feasibility of standard duration (12 sessions), CaCBTp in a lower middle-income country. We found that those receiving CaCBTp showed improvements in positive and negative symptoms and general psychopathology subscales of PANSS, as well as in insight when compared with TAU. At three months, reductions in PANSS general psychopathology, PANSS Positive Subscale, PANSS Negative Subscale, Total PANSS were statistically significant. Reduction in Delusion Severity and Hallucination Severity was also statistically significant, alongside improvements in insight scores. However, at six months, reduction in PANSS positive subscale was only found to be statistically significant. Nonetheless, this indicated sustained improvements at follow-up assessment at six months. These findings add to our initial work, and further suggest that this form of therapy is effective in this cultural group.

The judgment of CBT being effective in the treatment of psychosis is concluded from various meta-analyses [7]. Beneficial effects have been reported on positive and negative symptoms, along with mood, anxiety and social functioning [30]. Previous studies have shown that adapted forms of CBT are effective when used in minority populations [19, 29]. Our group has evaluated the use of CaCBTp in Pakistan, however, there is otherwise limited literature in this area.

The major limitation of this study is its sample size, the main aim being to establish feasibility of the intervention. We were unfortunately unable to collect qualitative data to inform the acceptability. A larger and appropriately powered study would be required in the future. Furthermore, brief CaCBTp has been shown to be effective [19], and comparison with standard duration therapy needs to be examined. Using the TAU group as a control is likely to over-estimate effect sizes, therefore, the results of this study must be interpreted with caution. Griner et al. (2006) used meta-analytic methodology to summarize data from 76 studies of culturally adapted psychological therapies [16]. The resulting random effects weighted average effect size was moderate (d = 0.45) [16]. The findings of our study would mirror this to a degree. Largely, these effect sizes are similar to those reported from non-adapted interventions [30]. More recent reviews determined that when controlling for masking, the effect sizes in non-adapted interventions became insignificant [7]. Scarcity of mental health services in lower-middle income countries, like Pakistan, is likely to contribute to the higher effect sizes. Similar findings have been reported in earlier randomised controlled trial of psychological intervention in Pakistan [31]. To control for this bias, future trials may consider the use of attention placebo controls like befriending or unstructured social support. Additionally, matching groups for confounders like duration of untreated psychosis and pharmacological treatment, would yield more reliable data for the efficacy of CaCBTp. Finally, this pilot study was conducted in Karachi, the largest city in Pakistan. It is a city with a relatively higher level of education and so adjustments would need to be made when applying this treatment to patients from a more rural population.

## Conclusion

This pilot study aimed to ascertain the feasibility of standard duration, culturally adapted CBT, for the treatment of psychosis. The feasibility of the treatment, has been demonstrated by high recruitment rates and the relatively low dropout rates in our intervention group. Our trial illustrates that it is feasible to offer CaCBTp as an adjunct to treatment as usual in patients with schizophrenia spectrum disorders, presenting to services in a low-income country. Prior to the implementation of this intervention, an adequately powered trial would need to be conducted to establish effectiveness of the intervention. Standard duration CaCBTp could be integrated into existing services; potentially being delivered by either mental health nurses or psychologists (Masters graduates, the equivalent of a UK Bachelor’s degree). Psychology is fast becoming a popular area of further education in Pakistan, however, following qualification many psychologists struggle to practice. This represents a significantly underused resource, and a feasible option for the implementation of CaCBTp. This study adds to our existing work, demonstrating that CaCBTp is effective in reducing psychopathology and improving insight. In future, it may be beneficial to compare brief and standard forms of therapy.
